# Recumbent cycling to improve outcomes in people with hip fracture: a feasibility randomized trial

**DOI:** 10.1186/s12877-021-02321-8

**Published:** 2021-06-29

**Authors:** Catherine M. Said, Marisa Delahunt, Andrew Hardidge, Paul Smith, Phong Tran, Luke McDonald, Emmanuel Kefalianos, Cathy Daniel, Sue Berney

**Affiliations:** 1 Physiotherapy, Melbourne School of Health Sciences, The University of Melbourne, Parkville, Australia; 2 Physiotherapy Department, Western Health, St Albans, Australia; 3 Australian Institute for Musculoskeletal Science (AIMSS), St Albans, Australia; 4 Physiotherapy Department, Austin Health, Heidelberg, Australia; 5 Orthopaedics, Austin Health, Heidelberg, Australia; 6 The University of Melbourne, Melbourne, Australia; 7 Department of Orthopaedic Surgery, Western Health, Footscray, Australia; 8 Nursing, Melbourne School of Health Sciences, The University of Melbourne, Parkville, Australia

**Keywords:** Hip fractures, Exercise, Bicycling, Feasibility studies, Early ambulation

## Abstract

**Background:**

Early mobilization after surgery is a key recommendation for people with hip fracture, however this is achieved by only 50% of people. Recumbent bike riding has been used in other populations with limited mobility and has potential to allow early exercise in people post hip fracture. The primary aim of this pilot trial was to demonstrate the feasibility of a trial protocol designed to determine the effect of early post-operative cycling in bed on outcomes in people with hip fracture.

**Methods:**

Single-blinded, multi-site randomized controlled pilot trial. Fifty-one people with hip fracture were recruited within 4 days of surgery from two sites in Victoria. Participants were randomly allocated to receive either usual care (*n* = 25) or usual care plus active cycling in bed (*n* = 26). The cycling intervention was delivered on weekdays until the participant could walk 15 m with assistance of one person. The primary outcomes were trial feasibility and safety. Clinical outcomes, including mobility (Modified Iowa Level of Assistance Scale) and delirium were measured at day seven post-operatively and at hospital discharge by an assessor blinded to group. Additional outcomes at discharge included gait speed, cognition and quality of life.

**Results:**

The intervention was safe, feasible and acceptable to patients and staff. Delivery of the intervention was ceased on (median) day 9.5 (IQR 7, 12); 73% of scheduled sessions were delivered; (median) 4 sessions (IQR 2.0, 5.5) were delivered per participant with (median) 9 min 34 s (IQR 04:39, 17:34) of active cycling per session. The trial protocol was feasible, however at day seven 75% of participants had not met the criterion (able to walk 15 m with assistance of one person) to cease the cycling intervention..

**Conclusion:**

In bed cycling is feasible post-operatively following hip fracture, however seven days post-operatively is too early to evaluate the impact of the cycling intervention as many participants were still receiving the intervention. A fully powered RCT to explore the effectiveness and cost efficiency of this novel intervention is warranted.

**Trial registration:**

The trial was prospectively registered (25/09/2017) with the Australian New Zealand Clinical Trials Registry ACTRN12617001345370.

## Background

Hip fracture is common and potentially life changing event. An estimated 296,000 people sustained hip fractures in the United States (US) in 2005 [1] and with the ageing population, it is anticipated numbers will increase. Hip fracture is a critical event in an older person’s life; it is estimated that 24% of people with hip fracture die within 12 months [2] and people with hip fracture have reduced function, health, quality of life and mental health [3]. Regaining the ability to walk following hip fracture is critical to short and long term outcomes [4–6]. Current evidence-based clinical practice guidelines from Australia and the United Kingdom recommend early assisted ambulation (walking) within 48 h of surgery, [7] however, this can be difficult to implement [8, 9]. People with hip fracture are typically older with complex health issues which can delay walking post-operatively. Walking can be resource intensive during the early post-operative phase, often requiring two people.

Alternative methods of exercising during the early-post operative period, while walking is difficult, may deliver benefits associated with exercise and improve walking and function. Studies have shown recumbent cycling is feasible and safe for other populations where walking is not a viable form of exercise, including patients in critical care units [10, 11]. Cycling may also impact on delirium, with one RCT reporting delirium was reduced from 57 to 33% of 104 patients in intensive care [12]. Thus, recumbent cycling has potential to both improve function and reduce delirium post-surgical management of hip fracture.

The primary aim of this pilot study was to demonstrate the safety and feasibility of a randomized controlled trial (RCT) protocol investigating whether early recumbent cycling improves post-operative outcomes in people with hip fracture. The secondary aim was to obtain data to assist with estimation of sample for a larger, multi-site RCT. It was hypothesized that it would be feasible and safe to deliver early exercise to people with hip fracture via a recumbent cycling program and that the protocol would be feasible.

## Methods

### Design

**Fig. 1:**
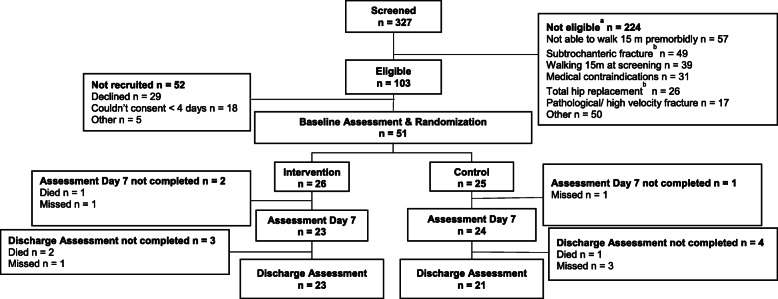
Participant flow through trial. a Some participants had more than one reason for ineligibility b. Exclusion criteria were amended during the trial (at *n* = 32 participants Site 1, *n* = 9 participants Site 2); these criteria were no longer a basis for exclusion

This was an investigator and assessor-blinded parallel group multi-site feasibility RCT. The trial was prospectively registered with the Australian and New Zealand Clinical Trial Registry (ACTRN12617001345370) on the 25/09/17, ethics and governance approvals were obtained prior to commencement and the study was conducted in accordance with all relevant guidelines and regulations. Written consent was obtained from the participant or person responsible prior to enrolment. Figure 1 provides an overview of trial design.

### Setting

In Australia, people are admitted to the acute hospital for surgical management (median length of stay of 7.6 days), with 49% transferred to a subacute hospital for rehabilitation [13]. Participants were recruited from the acute orthopedic wards at two major metropolitan health services in Victoria, assessments and interventions were conducted at acute and subacute sites at both health services.

### Participants

All people admitted to the acute orthopedic wards for surgical management of hip fracture were screened within four days of surgery; those who met the eligibility criteria were invited to participate by a member of the research team. While preferable to enrol people earlier post operatively (e.g 24 h), as staffing resources to support recruitment were limited, the window was extended to four days. This allowed participants and next of kin sufficient time to consider participation and maximise recruitment. Inclusion criteria were unable to walk 15 m with assistance at enrolment (within 4 days of surgery). Participants were excluded if: fracture was pathological or result of a high velocity trauma (eg car accident), other lower limb fractures were present, other medical conditions prevented mobilization/recumbent bike riding, weight bearing was restricted post-operatively, they were unable to walk > 15 m premorbidly, or they were not expected to survive seven days. Exclusion criteria were modified during the course of the trial and approved by the overseeing ethics committee. The novelty of this intervention initially dictated a conservative approach to participant selection. People with subtrochanteric fractures were excluded due to concerns about the suitability of cycling for this population and their exclusion from previous trials [14]. People who were managed with total arthroplasty were also excluded, as their management path may have differed from other people with hip fracture (people with hemiarthroplasty remained eligible for inclusion). These exclusion criteria were revised by investigators and removed (revised eligibility criteria implemented at site 1 on 16/11/18 when *n* = 32 recruited; implemented at site 2 on 02/01/2019 when *n* = 9 recruited). Anticipated discharge from the study health service in less than 7 days (including transfer to another hospital/ health service which could not provide the intervention or residential care facility) or weight over 135 kg (weight limit of equipment) were added as exclusion criteria on 01/08/2018 to avoid enrolment of participants unable to participate in the intervention or unlikely to complete the trial period (revised eligibility criteria implemented site 1 *n* = 24 recruited, site 2 *n* = 0 from). People likely to be transferred to a subacute facility within the health service remained eligible as they were able to continue participation in the trial.

### Randomization

A blocked, stratified randomization procedure allocated participants to the intervention group (cycling), or control group (no training). Participants were stratified based on study site and pre-existing dementia, defined as a documented history of dementia or an Informant Questionnaire of Cognitive Decline in the Elderly (Short Form) (IQCD) score > 3.3. Randomization was computer generated and performed by a third party. Allocation was concealed by sequentially numbered opaque envelopes, held in a secure filing cabinet. Once the baseline assessment was complete, the intervention therapist was provided with the corresponding envelope by a third party.

### Intervention

#### Usual care (intervention and control arms)

Both intervention and control groups received routine care which included nursing, medical and physical therapy. Care was initially provided in the acute hospital, but many participants were transferred to a rehabilitation ward. All participants received standard physical therapy, according to the current *Australian and New Zealand Guideline for Hip Fracture Care* [7] until the person was discharged from the study hospital or the treating team decided that there were no further rehabilitation goals. Data on physical therapy time and barriers to intervention were collected by the treating physical therapist.

#### Intervention (cycling) arm

In addition to usual care, participants in the intervention arm (cycling arm) actively cycled (with a passive back up rate of 5 cycles per minute) on a recumbent bike (MotoMed Letto 2) once a day on weekdays for up to 60 min, supervised by a physical therapist or physical therapy assistant not involved in delivery of usual physical therapy. While it was desirable to deliver the intervention on weekends, this was prohibited by limited funding for the study. Participants were encouraged to work at moderate intensity as measured by the BORG rating perceived exertion scale [15]. Participants continued the intervention until they met the mobility criterion of able to walk a minimum of 15 m with assistance of one person for two consecutive days, as assessed by the usual care physical therapist. This criterion was selected as clinical staff identified that once people could mobilise readily with one staff member, exercise could be integrated readily into functional activities (such as going to the toilet). The bike intervention was also ceased if the treating team (including medical staff, nursing staff and usual care physical therapist) determined rehabilitation should be discontinued as the person had no further rehabilitation goals (e.g. the person was not making gains and was unlikely to regain independent mobility). The physical therapist or physical therapy assistant who delivered the cycling intervention were not involved in assessment of mobility or any decisions about discontinuation of rehabilitation. Data on training time, intensity and barriers to training were recorded. Pain was assessed using a written numerical rating scale at the beginning and end of each session [16, 17]. The training protocol was amended (site 1 implemented 26/11/2018, *n* = 32 recruited, site 2 implemented 2/1/2019, *n* = 9 recruited) to suspend delivery of the cycling intervention if a participant’s International Normalized Ratio (INR) was > 3, to minimize the risk of increasing post-operative bleeding; the intervention recommenced once INR < 3.

### Outcomes

#### Feasibility outcomes

The primary outcome was feasibility and safety of early in-bed recumbent bike riding in people with hip fracture. Data on ***intervention feasibility*** included number of bike training sessions delivered, reasons for non-delivery of sessions, length of training sessions and feedback from patients on intervention acceptability. Data on ***intervention safety*** was collected by monitoring adverse events throughout the trial, including mortality, post-operative complications, issues with surgical fixation, bleeding, infections, falls, pressure areas and delirium. To ensure all adverse events were captured, data were collected by usual care physical therapists as they occurred and by blinded assessors via multiple sources, including medical records and hospital incident reports. Data on ***trial feasibility*** included participant recruitment and retention, completion of outcome measures, documentation of trial protocol deviations or variations, and feedback from trial staff about issues encountered in protocol delivery.

#### Clinical outcomes

Clinical outcomes were obtained by a research physical therapist blinded to group assignment and included core outcomes recommended for hip fracture trials [18].

The primary outcome was mobility 7 days post-surgery, measured using the Modified Iowa Level of Assistance Scale (mILOA). The mILOA is a 6 item functional measure that assesses level of assistance required to move from supine to sitting, sit to stand, walking, negotiation of a single step, walking distance and use of assistive device. It has demonstrated reliability and validity [19] and is responsive to change in people with hip fracture [20]. The time-point of 7 days post-surgery was selected as we anticipated the effect of this intervention would be greatest in the early post-operative period, when weight-bearing exercise such as walking is difficult. In addition, outcomes at this time point have been evaluated in other studies examining the effectiveness of exercise interventions during the early post-operative period [14, 20].

Additional secondary outcomes were measured at hospital discharge:

1. Mobility, measured using the mILOA and gait speed (assessed using the 6 m walk test),
2. Discharge destination,
3. Acute and subacute length of hospital stay (LOS),
4. Quality of life measured using the EuroQol 5D three level (EQ-5D-3L) and EuroQol Visual analogue scale (EQ-VAS) [21].
5. Cognitive status, assessed using the Montreal-Cognitive Assessment (MoCA) [22].

### Assessments

Three assessments were conducted by an assessor blinded to group assignment.

1. *Baseline Assessment* was conducted within four days of surgery. Data collected included participant age, gender, past medical and social history, premorbid medications, frailty [assessed using the Clinical Frailty Scale (CFS)] [23]), co-morbidities [assessed using the Charlson Co-morbidity Index (CCI)], [24] delirium (using the 3D Confusion Assessment Method instrument for research version 4.1) [25], fracture type, date and time of surgery, American Society of Anesthesiologists (ASA) score (obtained from the medical record), surgery type, anaesthetic type, intra-operative complications and other injuries. Premorbid dementia/ cognitive decline was determined by documentation in the medical record or a score > 3.3 on the Informant Questionnaire of Cognitive Decline in the Elderly (Short Form) (IQCD) [26]. Premorbid mobility was assessed using the New Mobility Score (NMS) [27].
2. *Day 7 Post-Surgical Assessment* was conducted on the nearest usual business day seven days post-surgery. This was the primary trial endpoint. If the participant was discharged from the study hospital prior to this assessment, data were collected on discharge. The primary outcome measure (mILOA) and delirium (3D CAM instrument for research (version 4.1) were assessed.
3. *Discharge Assessment.* This assessment was conducted within 48 h of discharge. Outcomes including mILOA, gait speed (6 m walk test), EQ-5D-3L and EQ-VAS, MoCA, discharge destination and acute and sub-acute LOS. Assessment of cognitive status at discharge minimized the impact of acute delirium on test performance.

In addition the usual care physical therapist conducted daily assessments on weekdays for a minimum of 7 days post-surgery and continued daily assessments until the participant walked a minimum of 15 m with one person, two days in a row. These included assessment of delirium, (assessed by the SHORT CAM) [28] pain, assessed using a written numerical rating scale, [16, 17] and distance walked during the physical therapy session.

Participants in the intervention arm provided feedback after the final intervention session via a semi-structured interview. This was conducted by a staff member not involved in the person’s care or in other aspects of the trial.

### Sample size

As this was a feasibility trial formal sample size estimation was not completed. Based on experience with other trials, [29] a sample of 60 participants (30 each arm) was anticipated to provide sufficient data to evaluate the feasibility of the intervention and protocol.

### Blinding, contamination and monitoring

Participants and intervention therapists could not be blinded to group assignment. Group assignment was not disclosed to usual care physical therapists or assessors. Blinding was evaluated by asking staff to which group they thought a participant was assigned. Usual care staff were asked at the end of the intervention phase; assessors were asked at each assessment.

### Data analysis

To determine intervention feasibility, the proportion of scheduled sessions delivered and median length of session time were calculated and reasons for non-delivery of sessions inspected by the investigators. Patient feedback was examined thematically. To determine intervention safety, adverse events were collated and inspected.

To determine trial protocol feasibility, data on key project quality indicators were collated, including data on participant recruitment (including time to recruitment), retention and completed outcome data at each time point. Trial protocol deviations or variations and trial staff feedback was documented and reviewed by investigators.

Baseline characteristics were inspected for the intervention and control groups. If data were missing, a blinded assessor reviewed the medical record notes to determine the mILOA score. Data for remaining outcomes were recorded as missing. Primary and secondary outcome data were examined for normality, and descriptive data (median, IQR or frequencies) calculated. As this study was not sufficiently powered, tests for significance were not planned.

## Results

### Participant recruitment and retention

Fifty-one participants were recruited between 14 November 2017 and 29 January 2019, with the final discharge assessment completed on 18 February 2019. Recruitment at site 1 was suspended on three occasions due to equipment breakages. Forty-two participants were transferred to a subacute facility within the health service during the trial (median day of transfer day 6 IQR 4.8, 8), an additional three participants were discharged from the trial to a subacute facility outside the health service. Trial flow is presented in Fig. 1 and baseline characteristics in Table 1. Participants assigned to the intervention arm (*n* = 26) tended to have better premorbid mobility (higher scores on the NMS) and were less frail (lower scores on the CFS). Thirty-three baseline assessments were completed within two days of surgery; remaining assessments within four days.

**Table 1:** Characteristics of participants at baseline

	Bike (***n*** = 26)	Control (***n*** = 25)
Age	82 (71, 88)	85 (80, 89)
Gender (Male) [n (%)]	8 (31%)	7 (28%)
Days surgery to assessment	2.5 (2, 3)	2.0 (1, 2)
New Mobility Scale	7.5 (5, 9)	6 (5, 9)
Clinical Frailty Scale	4 (3, 5)	5 (3, 6)
Charlson Co-morbidity Index	1 (0, 2)	2 (1, 4)
Cognitive impairment (yes) [n (%)]	14 (54%)	14 (56%)
Delirium (yes) [n (%)]	15 (58%)	13 (52%)

Median and interquartile range (IQR) reported unless otherwise stated

### Data quality

Complete baseline data were available for all participants. Data were not collected for three participants at day seven (see Fig. 1). Four day seven assessments were completed early (*n* = 3 day 3, *n* = 1 day five) and at the same time as the discharge assessment because the participant was discharged from hospital. In addition, delirium was not assessed at day seven for two participants (intervention arm). Data were not collected for seven people at discharge (see Fig. 1). Data were missing for gait speed for another 10 participants (four assessments not completed, six participants physically unable to complete the test), delirium for one participant, MoCA for 13 participants, EQ-5D-3L for 5 participants and EQ-VAS for 8 participants.

Data quality for usual care and cycling intervention physical therapy sessions are presented in Table 2. No assessments of delirium were performed within the first seven days for three participants in the intervention arm and two participants in the control arm.

**Table 2:** Data quality for usual care physical therapy sessions and cycling intervention sessions

	Usual Care Sessions	Cycling Sessions
Records collected (n)	400	150
Sessions delivered (n, %)	324 (81%)	109 (73%)
Mobility status ^a^ (n, %^b^)	324 (100%)	N/A
Session length ^a^ (n, %^b^)	307 (95%)	103 (94%)
Time on bike ^a^ (n, %^b^)	N/A	108 (99%)
Activity time ^a^ (n, %)	N/A	102 (94%)
Distance cycled ^a^ (n, %)	N/A	106 (97%)
Borg scores ^a^ (n, %^b^)	175 (54%)	102
Pain scores ^a^ (n, %^b^)	238 (75%)	108
Delirium ^a^ (n, %^b^)	277 (85%)	N/A
Delirium assessments first 7 days ^a^ (median, IQR)	2 (2–3)	N/A

N/A = not applicable

^a^ Number of records with data on the variable documented (e.g. mobility status was documented on 324 usual care session records.) Mobility status and delirium were only documented during usual care physical therapy sessions. Time on bike, activity time and distance cycled were only recorded during the cycling intervention sessions

^b^Percentage calculated based on sessions delivered

### Intervention delivery & protocol compliance

**Fig. 2:**
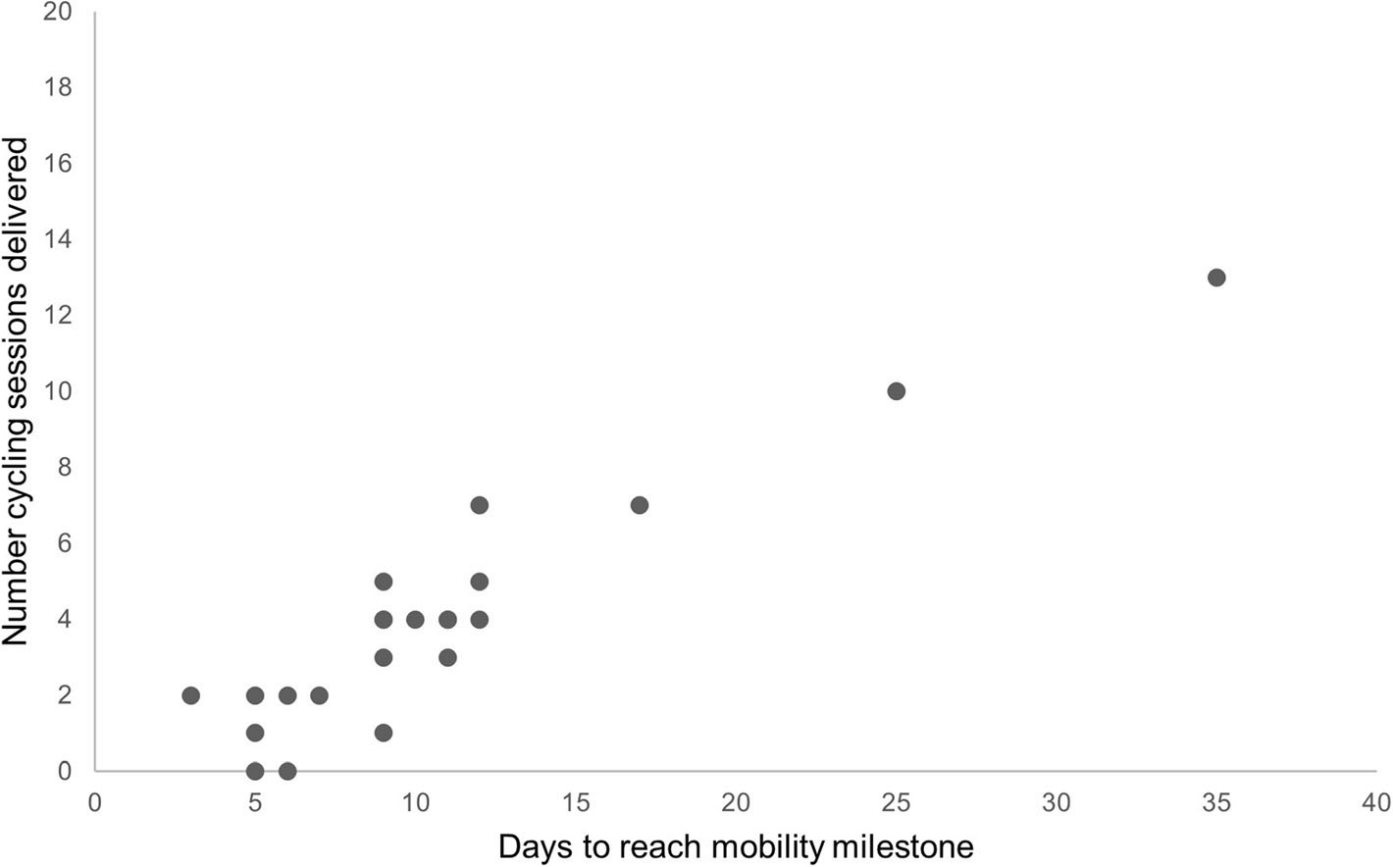
Scatterplot of number of delivered cycling intervention sessions versus days to reach mobility milestone. Each dot represents a participant in the intervention (cycling) arm of the trial

Two people in the intervention arm did not receive any cycling intervention as they met the criterion for ceasing the intervention before delivery of the first session (baseline assessments were completed on Friday, first cycling intervention session scheduled Monday). Forty-one scheduled cycling intervention sessions (27%) were not delivered. Reasons for missing sessions were participant unwell (10 sessions), staff availability or public holidays (11 sessions), participant declined (6 sessions), participant transfer between sites (4 sessions) or equipment problems (2 sessions). The number of cycling intervention sessions each participant received is illustrated in Fig. 2, with a median of 4 sessions (IQR 2.0, 5.5) delivered. Fifty percent of sessions were delivered between days one to seven post-operatively, 29% were delivered between days eight to fourteen, 15% were delivered between days 15 to 21 and 6% were delivered more than 21 days post-operatively. Median session length was 12 min 59 s (IQR 10:14, 18:15), of which 9 min 34 s (IQR 04:39, 17:34) was active cycling and rode a median of 1.56 km (IQR 0.72, 2.66). Intervention delivery ceased on day 9.5 (IQR 7, 12), when participants achieved the mobility milestone. The median Borg score was 13.0 (IQR 11, 13.5) and median change in pain score was 0 (IQR -1.0, 1.5).

### Safety

Details of adverse events reported are provided in Table 3. Two serious adverse events occurred during the intervention phase. One participant in the intervention arm developed chest pain while cycling on the bike. The intervention was ceased and routine hospital procedures were followed. One participant in the intervention arm developed a wound infection secondary to a hematoma due to hypercoagulation. The infection did not respond to antibiotics and hip washout, thus the prosthesis was removed and replaced with an antibiotic spacer. Medical staff were consulted and the intervention was not delivered while there were clinical signs of hematoma or infection. The intervention protocol was modified to suspend delivery of the intervention if a person’s INR was > 3. One person in the control arm had a periprosthetic fracture after the intervention phase.

**Table 3:** Adverse events from baseline assessment to discharge assessment

	Bike (n = 26)	Control (n = 25)
Death	2 (8%)	1 (4%)
ICU admission	0 (0%)	3 (16%)
MET call	6 (23%)	10 (40%)
Return to theatre	1 (4%)	0 (0%)
Transfer to acute hospital	1 (4%)	2 (8%)
Cardiac Event	1 (4%)	3 (16%)
Bleeding	3 (12%)	1 (4%)
Low HB	8 (31%)	8 (32%)
Hypotension	11 (42%)	8 (32%)
DVT	0 (0%)	0 (0%)
PE	2 (8%)	0 (0%)
Issues with fixation	0 (0%)	1 (4%)
Chest Infection	2 (8%)	10 (40%)
Urinary Tract infection	2 (8%)	0 (0%)
Other infection	3 (12%)	0 (0%)
Fall	4 (15%)	4 (15%)
Pressure area	1 (4%)	2 (8%)
Delirium		
Within first seven days^a^	5 (22%) (*n* = 23)	8 (35%) (n = 23)
Day 7^b^	6 (30%) (n = 20)	11 (48%) (n = 23)
Discharge^b^	8 (36%) (n = 22)	8 (38%) (*n* = 21)

MET = Medical Emergency Team call

a Assessed by usual care staff

b Assessed by blinded assessor

### Blinding and contamination

Assessors indicated they remained blinded for 88% of the seven day assessments and 86% of the discharge assessments. Blinded assessors correctly identified group assignment for 72% of the day seven assessments (kappa .450, *p* = .002) and 65% of the discharge assessments (kappa .321, *p* = .032). When assessors who identified as unblinded were removed from analysis, assessors correctly identified group assignment for 68% of day seven assessments (kappa .384, *p* = .013) and 60% of discharge assessments (kappa = .215, *p* = .180).

Participants in the intervention and control groups received a similar number of usual care physical therapy sessions [intervention median 6 (IQR 3,7); control 6 (IQR 4, 7)]and session lengths were comparable [intervention median 26 (IQR 20, 30); control 28 (IQR 20,30)].

### Participant and therapist feedback

Fourteen participants provided feedback on their experience on the bike and in the study. Twelve indicated they were ‘satisfied’ or ‘very satisfied’ with their overall experience exercising on the bike, one person was ‘neither satisfied nor dissatisfied’ and one person was ‘dissatisfied’. Things participants liked about using the bike included ‘(the bike) helped move the leg’, was ‘easy to use’, ‘helped with progress’ and gave them a ‘sense of achievement’. When asked what they liked least, one participant reported the physical therapist encouraged them to do more than they felt could do, one participant indicated she preferred walking and one reported they felt ‘off balance’. Remaining participants either had nothing they disliked or indicated they preferred to use the bike more.

Therapists involved in delivering the intervention indicated they liked using the bike and some reported it was easier to use than they anticipated. Staff indicated good communication between members of the research team (usual care staff, intervention staff, blinded assessors and investigators) was critical to project success; including daily communication and regular formal meetings. One challenge identified was staffing resources, including staff turnover and absences, and the importance of having sufficient people trained to complete tasks was highlighted. Staff identified that it was difficult to maintain blinding of usual care staff. Ensuring completion of data collection forms by usual care staff was also identified as a challenge. Several staff identified it was challenging incorporating trial duties into their clinical workload. Benefits of being involved in the study included learning new skills; such as experience with the bike, new outcome measures and project management. Some staff reported teamwork and communication improved due to trial involvement and satisfaction working on a project that complemented clinical work.

### Clinical outcomes

Outcomes at day seven and discharge are provided in Table 4. Mobility as assessed by the mILOA was similar between the two groups at day seven and discharge. Participants in the intervention arm reached the mobility milestone slightly earlier.

**Table 4:** Outcomes at day seven and hospital discharge (median, IQR)

	Bike (n = 26)	Control (n = 25)
**Seven day assessment**		
mILOA	21 (16, 29) (n = 25)	21 (18, 27)
**Discharge Assessment**		
mILOA	8 (6, 13) (n = 24)	9 (6, 15) (n = 24)
Gait Speed (m/s)	0.33 (0.19, 0.50)^a^ (*n* = 19, 73%)	0.47 (0.22, 0.58)^b^ (*n* = 15, 60%)
MoCA	18 (11, 26) (*n* = 17)	15 (11, 20) (*n* = 14)
EQ-5D-3L	.62 (.52, 76) (*n* = 21)	.61 (.33, .71) (*n* = 18)
EQ-VAS	62 (50, 80) (n = 18)	53 (9, 80) (n = 18)
**Time to reach mobility milestone (days)** ^c^	9.0 (6.0, 12.0) (*n* = 22)	10.5 (6.8, 14.0) (n = 22)
**Acute LOS**	6.0 (4.0, 7.3)	6.0 (5.0, 9.5)
**Subacute LOS**	22.5 (13.3, 29.8) (*n* = 20)	17.5 (12.8, 29.5) (n = 22)
**Total LOS**	20.5 (11.5, 36.3)	23.0 (13.0, 36.5)
**Discharge destination**		
Home	15	13
Residential care	6	8
Other	5	4

mILOA = Modified Iowa Level of Assistance Scale; MoCA = Montreal-Cognitive Assessment; EQ-5D-3 = EuroQol 5D three level; EQ-VAS = EuroQol Visual analogue scale; LOS = length of stay

a. Two participants were unable to perform the test, six participants did not complete the assessment (two died, two no discharge assessment, two no gait assessment)

b. Four participants were unable to perform the test, six participants did not complete the assessment (one died, three no discharge assessment, two no gait assessment).

c. Two people died prior to reaching milestone (one control, one intervention). Five people did not achieve the milestone before discharge (two control, three intervention)

## Discussion

It is feasible and safe for people with hip fracture to exercise using an in-bed recumbent bike in the early post-operative period. Participants commenced using the bike typically less than 3 days post-surgery and over 70% of scheduled sessions were delivered, comparable to the delivery rate of usual physical therapy sessions (Table 2). Participants exercised for nearly 10 min at moderate intensity and pain was not increased with cycling. Feedback from participants assigned to the intervention arm indicated the bike was acceptable to people with hip fracture. We also demonstrated that while many aspects of the protocol were feasible, some modifications would be required to run a large and rigorous multisite trial.

There was no overall increase in adverse events in the intervention group; however two adverse events occurred while participants were in the intervention phase. One was a cardiac event; while it may have been related to exertion, the risk of this occurring is similar to risks in traditional forms of exercise such as walking. The second event was development of a hematoma and subsequent wound infection. While this was determined to be due to hypercoagulation, it is possible that exercise may exacerbate bleeding. Therefore the protocol was modified and we recommend in-bed bike riding is not performed if a person’s INR is > 3.

Dosage of the cycling intervention varied between participants. The rationale behind the intervention was to facilitate early post-operative exercise in people with limited mobility to reduce functional decline and facilitate faster recovery of function. Thus, the cycling intervention was ceased once the participant achieved the functional criterion of walking 15 m with assistance of one person for two consecutive days. As indicated in Table 3, the time taken to meet the mobility milestone varied; with IQR from day 6 to day 12. This led to variation in the number of sessions received; participants who took longer to regain mobility and who were most at risk of functional decline had the opportunity to receive more sessions than those who regained mobility quickly. As the cycling intervention had not previously been delivered to people with hip fracture in the early post-operative period, we did not have a predetermined target dosage (e.g. number of sessions, length of session). However, it is likely the dosage was suboptimal in the early post-operative period; the first intervention session was delivered a median of 3 days (IQR 2, 4) post-surgery and the intervention was not delivered on weekends, due to funding limitations. To maximise dosage and any potential benefit of the intervention, future trials should aim to commence the intervention within 24–48 h of surgery and provide access to intervention seven days a week.

Recruitment to clinical trials is recognised as being particularly challenging in acute orthopaedic settings and trials involving older people [30, 31]. As illustrated in Fig. 1, nearly two thirds of people screened were not eligible. As the intervention was novel for this population, we initially had a conservative approach to eligibility. Earlier inclusion of participants with either a subtrochanteric fracture or total hip replacement would have increased the number of people eligible to over 50%. It is reasonable to exclude people not able to walk pre-morbidly, (17% of those not eligible) from a trial to improve mobility. Similarly, people who quickly regain mobility (12% of those not eligible) are also unlikely to benefit from this intervention. Approximately 50% of those eligible consented to the study. Obtaining consent in time critical situations can be challenging [30, 31], which is why we extended the recruitment window to 4 days. Despite this, it was not possible to obtain consent within four days for over a third of those who did not consent, mainly due to limited staff time. Adequate staffing and processes to support timely recruitment to any future trials are essential.

Maintaining blinding of usual care physical therapists was difficult due to the physical presence of the bike. While we attempted to implement strategies to reduce this (e.g. providing intervention sessions when usual care staff were not on the ward) this proved impractical. Monitoring delivery of usual care demonstrated there were no differences between groups. However, in future trials it is essential to monitor usual care delivery to detect contamination and ensure the robust completion of the trial. We also found blinding of assessment staff was not always maintained; assessors reported they become ‘unblinded’ in 12–14% of assessments. Even when assessors reported they remained blinded, results indicated they identified group assignment more likely than anticipated by chance. We used several strategies to optimize blinding; assessors did not work on wards where trial participants were cared for and participants were cautioned not to discuss treatment with assessor. Nonetheless, results suggest additional strategies to maintain blinding are required and emphasise the importance of monitoring the adequacy of assessor blinding in future trials.

While generally data quality was high, several issues would need to be addressed in a larger trial. Assessments were missed at day seven and discharge. Discussion with staff identified factors contributing to missed assessments included temporary and unplanned absences of key project staff and participant movement from acute to subacute hospitals. At discharge assessment, MoCA and EQ-5D-3L data were missing for a high proportion of participants and feedback was only obtained from 54% of people who used the bike due to participants declining to complete these components, lack of time (either participant or staff), or cognitive impairment. Documentation of session length and ambulation status by usual care staff was generally completed, however recording of pain and Borg scores was less complete, with cognitive impairment again a reason for non-completion. In contrast, completion rates of pain and Borg scores during intervention sessions were relatively high; suggesting other issues such as staff training or workload may have also impacted on completion rates in usual care sessions. Delirium was generally only assessed on two or three days within the first seven days and assessments were generally not completed on weekends. This makes identification of delirium via the usual care assessments sub-optimal, thus strategies to improve identification of delirium on a daily basis would need to be considered for future trials.

The study was not powered to detect an effect on clinical outcomes; however it is of interest to explore clinical outcomes of participants in the intervention and control arms. At seven days post-surgery mobility, measured using the mILOA, was similar between groups. However, most participants in the intervention (cycling) arm had not yet completed the intervention; with the median time of 9 days to achieve the mobility milestone (walking 15 m with assistance of one person for participants for two consecutive days). This suggests day seven is too early to measure the full effect of a cycling intervention. The intervention group achieved the mobility milestone one day earlier and was discharged two days earlier, although findings may be influenced by better premorbid mobility and less frailty in the intervention group. Other outcomes, including delirium, gait speed, MoCA and quality of life, must be interpreted with caution due to incomplete data. Gait speed was faster in the control group, which may indicate poorer outcomes in the cycling group. However fewer people in the control group completed assessment of gait speed. The proportion of people with delirium, as assessed by usual care therapists and the blinded assessor at day seven, was lower in the intervention group. There is evidence that in-bed bike cycling impacts on delirium in patients in critical care; thus it will be important to monitor delirium in future trials. A fully powered study is required to fully evaluate whether in-bed cycling improves outcomes in people with hip fracture.

Based on results of this feasibility trial, several protocol modifications would be recommended for future trials. In future trials, participants should be recruited within 24 h of surgery and interventions should be available on weekends to maximize exercise dosage. In addition, as previously discussed, as many people had not completed the cycling intervention by day seven, this time point is too early to evaluate the full effect of the intervention. Inspection of the interquartile ranges indicate that 75% of participants reached the mobility milestone by day 12 to day 14; this suggests this time point may be more appropriate. The future trial should consider strategies to improve daily assessments of delirium and assessor blinding. Staff feedback highlighted the importance of local project management practices and robust communication strategies, particularly as most participants were transferred between acute and subacute facilities. This feasibility study was completed with limited funding; future studies will require adequate funding to appropriately support staff in project management, intervention delivery and assessment. Finally, equipment breakages required suspension of study recruitment on three occasions thus future trials should consider contingency planning for equipment failure.

## Conclusion

In-bed recumbent cycling is feasible and safe for people with hip fracture. While generally the proposed RCT protocol is feasible, refinements are required to optimize intervention dosage and ensure measurement of outcomes at the most appropriate time point to ensure successful completion of a high quality trial.

## Data Availability

The datasets generated and/or analysed during the current study are not publicly available but are available from the corresponding author on reasonable request.

## Abbreviations

ASA: American Society of Anesthesiologists
CAM: Confusion Assessment Method
CCI: Charlson Co-morbidity Index
CIs: Confidence Intervals
(EQ-5D-3L): EuroQol 5D three level
(EQ-VAS): EuroQol Visual analogue scale
HR: Hazard ratio
IQCD: Informant Questionnaire of Cognitive Decline in the Elderly (Short Form)
INR: International Normalized Ratio
IQR: Interquartile range
LOS: Length of hospital stay
mILOA: Modified Iowa Level of Assistance Scale
MoCA: Montreal-Cognitive Assessment
NMS: New Mobility Score
RCT: Randomized controlled trial
